# Medical Science Data Value Evaluation Model: Mixed Methods Study

**DOI:** 10.2196/63544

**Published:** 2025-08-21

**Authors:** Dandan Wang, Yaning Liu

**Affiliations:** 1 Business School, Henan University of Science and Technology, Luoyang, China; 2 School of Economics and Management, Guangzhou University of Applied Science and Technology, 20 Fengle Road, Lianhua Town, Dinghu District, Zhaoqing, Guangdong Province, 526000, China, 86 13015598823

**Keywords:** value evaluation model, medical informatics, open platform, health medicine, scientific data

## Abstract

**Background:**

Medical science data hold significant value, and open platforms play a crucial role in unlocking this potential. While relevant platforms are being developed, the overall usage of these data values remains limited.

**Objective:**

This study aims to propose a set of practical and effective data value evaluation processes and methods for medical science data open platforms, enabling them to manage and unlock the value of these data.

**Methods:**

Integrating the information system success model, technology acceptance model, and consumer perceived value theory, a set of medical science data value assessment index systems was developed by adopting the literature review and expert survey methods. Data from 10 domestic and international open platforms were collected and empirically analyzed using the entropy-weighted Technique for Order Preference by Similarity to Ideal Solution technique.

**Results:**

Based on the scores of each indicator, the intragroup correlation coefficient was calculated to be 0.489, indicating consistency in the evaluation. The highest information entropy values and weights determined using the entropy weighting method were the number of datasets (0.70, 17.68%), data timeliness (0.77, 13.44%), search comprehensiveness (0.78, 12.92%), and system responsiveness (0.80, 11.55%), respectively. Based on the weighted analysis, the platform with the highest overall score was the National Population Health Sciences Data Center, with a score of 62.32.

**Conclusions:**

The evaluation index system and model developed can be used not only to optimize the platform’s data value evaluation processes, but also to enhance the platform’s overall data value and encourage users to reuse data.

## Introduction

### Background

Medical science data refer to data generated through basic and applied research, experimental development, as well as raw and derived data obtained through observation, monitoring, inspection, and testing [1]. These data include electronic health records, long-term treatment outcome data, experimental drug performance data, and wearable device data. Medical science data are considered a strategic resource, offering significant scientific benefits such as reducing research costs, accelerating the research process, improving research quality, and increasing the impact of data [2]. For example, electronic health records can not only help doctors make diagnostic and treatment decisions more accurately and quickly, but also assist patients in understanding their own health status and promoting self-management.

According to investigations, approximately 469 and 750 medical science data platforms have been registered on FAIRsharing and Re3data, respectively [3,4]. Despite such widespread availability, user survey reports indicate that the usage and downloads of many platforms are far lower than expected. This gap stems from users’ insufficient awareness of the research field and potential benefits. For example, clinicians still rely heavily on experience and traditional methods for clinical decision-making rather than using data-driven analytical tools. The public perceives medical data as overly specialized, thus overlooking its importance. Platforms often fail to demonstrate the value of data in an understandable and user-friendly way, making it difficult for users to grasp its practical applications.

Existing open health care platforms have challenges such as limited open sharing of data, low standardization, poor data quality, insufficient value mining, insufficient privacy protection, and low usage. The main reason behind these challenges is the inability of both platforms and users to objectively quantify the potential value of data. Platforms suggest identifying the key factors in the formation of data value and allocating resources effectively, while users are unable to carry out data activities based on the true value of data.

### Prior Work

In the past, scholars have assessed the value of data in different scenarios, including the assessment of enterprise data value in business contexts [5,6], the evaluation of government data [7,8], and social media data [9] in nontransactional scenarios. Given that platform data are more readily accessible, research has increasingly focused on assessing the value of data from platforms. In terms of assessment theories and perspectives, assessment is usually conducted from the accounting perspective, stakeholder perspective [10], data life cycle or data value chain perspective [11], data traceability perspective [12], and seldom adopts a comprehensive perspective or innovative theories. In terms of evaluation content and methods, the valuation of intangible assets [13,14] typically uses approaches such as the cost method, market method, and income method, with a focus on data value components such as cost and income. In recent years, scholars have introduced revenue monetization, impact-based approaches, experimental and survey techniques, comprehensive multiattribute evaluation, and intelligent methods, which emphasize evaluation from multiple value dimensions and factors affecting data value [15-17].

Medical science data are rich and sensitive enough to generate valuable scientific insights, but the assessment of their value remains fragmented. Initially, Wang and Strong [18] proposed one of the most widely used frameworks for assessing data quality, but due to the complexity of the data quality concept, there is still no consensus on its measurement. Given that different types of data apply to different contexts, they cannot be assessed with a one-size-fits-all approach [19]. Scholars have made significant efforts to assess the value of research data. For example, Schmidt et al [20] developed a framework for assessing the quality of research data. Uribe et al [21] assessed the quality of research data based on the FAIR (Findable, Accessible, Interoperable, and Reusable) principle. Regarding the quality of health care data or information, Feder [22] summarized the dimensions and methods for assessing the quality of EHR data. Kim et al [23] proposed a conceptual model for the quality of health care data. Zhang and Trace [24] explored the quality of self-tracking data. Lasim et al [25] identified accuracy, timeliness, completeness, and consistency as key dimensions for assessing the quality of health care data. Wu et al [26] described the quality of health data in terms of dataset quality, descriptive data quality, and metadata quality. This study assesses the value of medical science data on open platforms based on an integrated perspective and using a combination of qualitative and quantitative methods, aiming to fill the gaps in existing research.

### Objective

This study aims to construct the index system and model of medical science data value assessment from the perspective of the platform and propose the optimization process of data value assessment and data value enhancement strategy. Specifically, the medical science data open platform includes a medical science data resource base and a knowledge base. The platform perspective refers to analyzing and understanding the role, value, and function of the platform in a specific ecosystem from the perspectives of the platform’s overall architecture, functional design, and interaction with users, data, and other stakeholders.

The questions to be addressed are as follows: (1) How to construct the medical science data value assessment index system and assessment model? (2) How much do different assessment dimensions and indicators contribute to the value of medical science data? (3) How scientific and usable are the constructed assessment models?

## Methods

### Study Design

**Figure 1.**
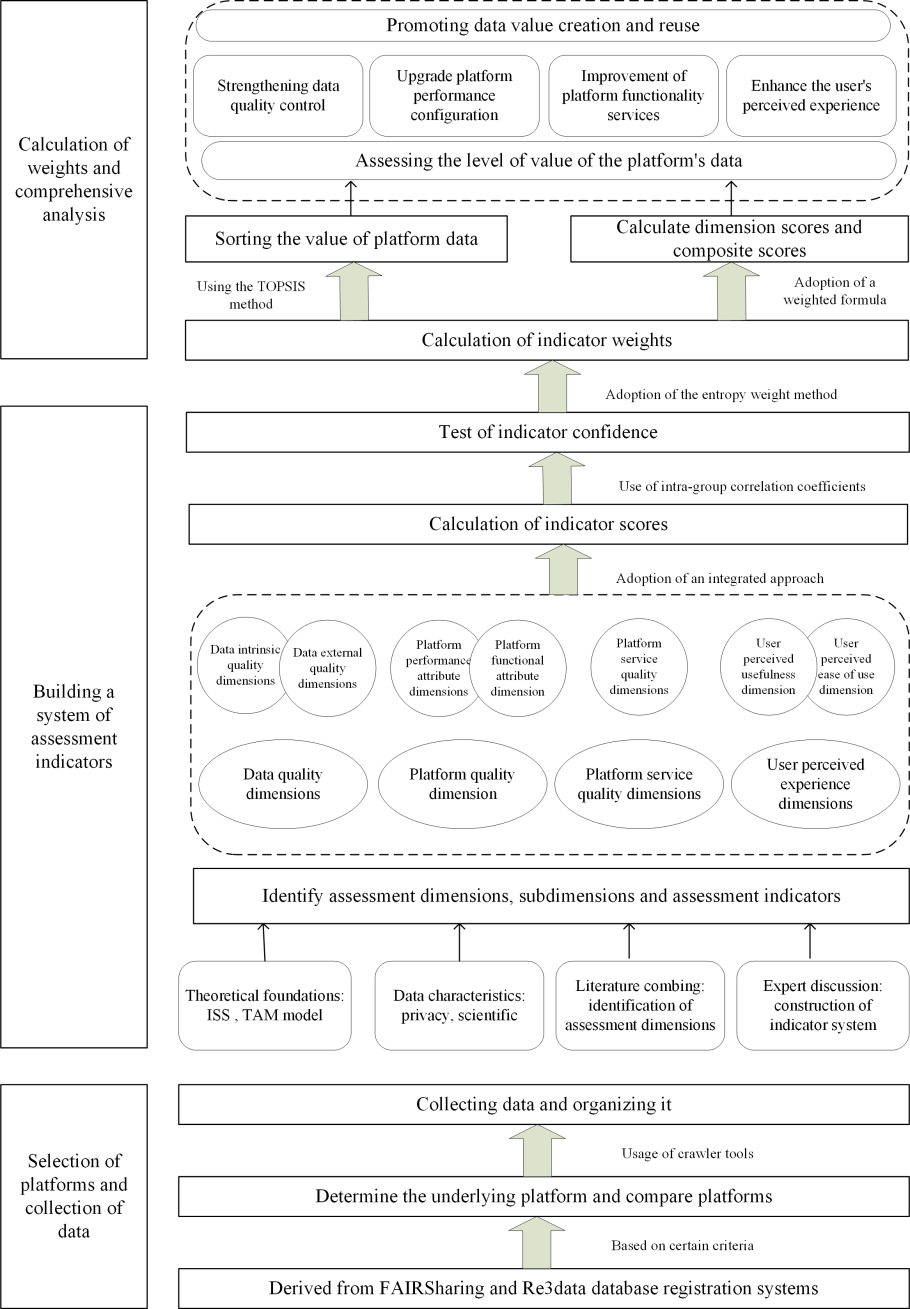
Model for assessing the value of medical science data. TOPSIS: Technique for Order Preference by Similarity to Ideal Solution.

The key steps in this process include developing an assessment index system, calculating weights, and conducting comprehensive analyses, which are as follows:

1. Determination of the assessment subject: a selection of domestic and international platforms was chosen as subjects for comparison, using FAIRsharing and Re3data as primary sources, with large datasets serving as standards. These data resources were mined using web scraping tools to provide the foundation for calculating indicator weights.
2. Development of the assessment indicator system: drawing on the theoretical model, a review of the literature, and the characteristics of the data, the initial set of assessment indicators was identified. The indicator system was refined through expert evaluation, with manual verification and other methods used to assess indicator scores and validate their reliability using the intraclass correlation coefficient (ICC).
3. Collection of indicator weights: the entropy weight method was used to determine the weights of the indicators. For a comprehensive evaluation, the Technique for Order Preference by Similarity to Ideal Solution method was applied. The weighted scores were then used to calculate the overall data value of each platform. This model aids platforms in quantifying the potential value of their data, assessing data quality, and enhancing control mechanisms. The study design is presented in Figure 1.

### Construction of the Indicator System

**Figure 2.**
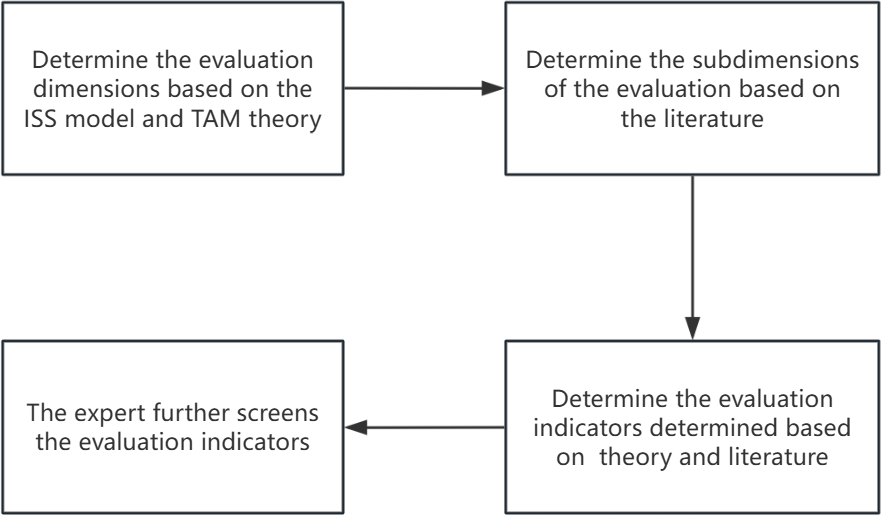
Steps to construct an evaluation value indicator system. ISS: information systems success; TAM; technology acceptance model.

Figure 2 shows the steps to build the evaluation index system.

#### Assessment Framework Construction

The formation of the potential value of the data mainly relies on the platform, and the user is the platform experiencer and data user, who will develop specific subjective cognitions while using the platform data and will give an overall evaluation of the platform’s data value. As a result, medical science data value can be defined as the perceived value generated by users in the process of discovering, acquiring, manipulating, and reusing medical science data captured and organized by the platform, involving data, platform, and user dimensions. The data dimension directly assesses data value, while the platform and user dimensions indirectly assess data value.

This study constructs an evaluation theoretical framework based on the information systems success model (ISSM), the technology acceptance model (TAM), and the theory of perceived value of users. An open platform can be regarded as an information system, so the influence factors of data value realization can be analyzed using ISSM. However, because ISSM focuses on analyzing the influence of the data and platform dimensions on the user’s net benefit (perceived value), it is integrated with TAM to supplement the influence of the user dimension on the user’s net benefit.

**Figure 3.**
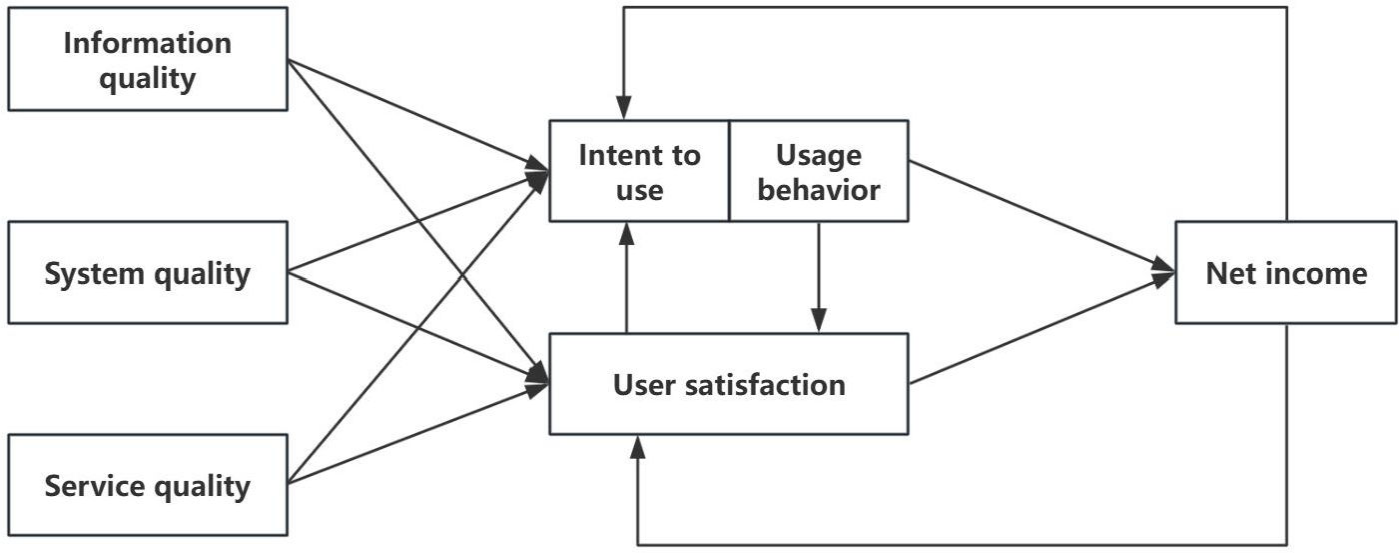
Information systems success model.

As shown in Figures3  4, in ISSM, 3 factors—information quality, system quality, and service quality—affect users’ willingness to use. In TAM, the user’s perceived usefulness and perceived ease of use will also affect the user’s attitude. Therefore, the dimensions of information quality, system quality, service quality, perceived usefulness, and perceived ease of use were selected to evaluate the value of data.

**Figure 4.**
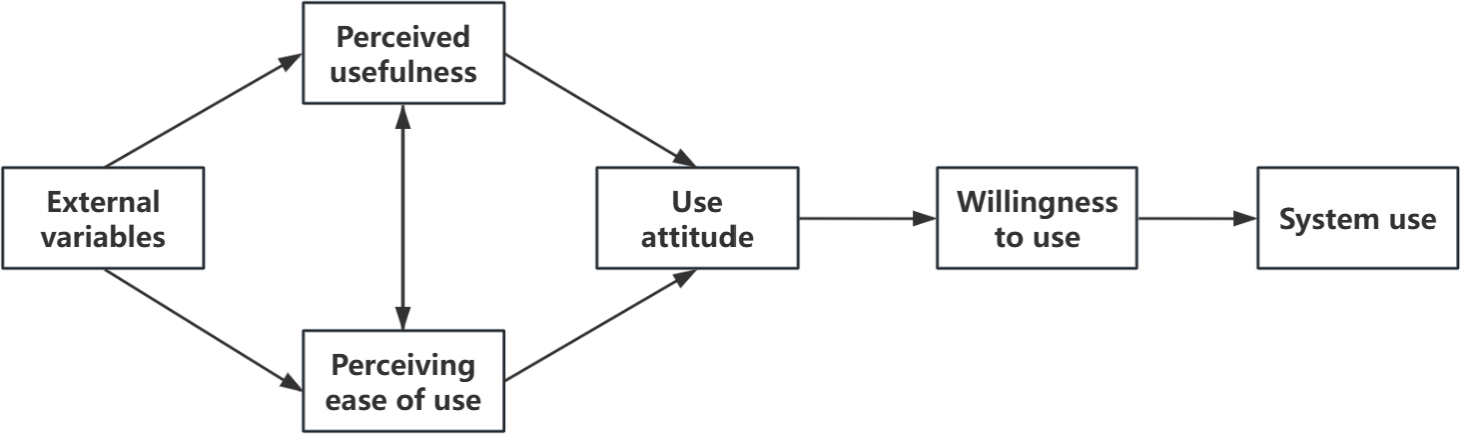
Technology acceptance model.

Table 1 illustrates the dimensions of each theory and their unique role in the model.

In this framework, good data quality ensures that users have access to high-quality data resources; good platform quality and platform service quality ensure access to perfect infrastructure and services; and perceived usefulness and ease of use ensure motivation to use the data. All 5 dimensions influence users’ willingness to use the system and their behaviors, which stimulates the formation of data value.

**Table 1.** Theoretical dimension action table.

Theoretical model	Assessment dimensions	Unique role
ISSM^a^	Information quality: data quality System quality: platform quality Quality of service: platform service quality	Evaluate the objective attributes of the data (eg, accuracy and completeness), stability of the technology platform, and effectiveness of support services.
TAM^b^	User-perceived usefulness User-perceived ease of use	Measure the user’s subjective perception of the value of the data for their research or practice, and the impact of the platform’s ease of use on willingness to use.
Consumer-perceived value theory	Final value assessment	Integrate objective quality and subjective perception to form the user’s overall judgment of the value of the data (eg, willingness to pay and willingness to recommend).

a ISSM: information systems success model.

b TAM: technology acceptance model.

#### Selection of Assessment Indicators

Based on the principles of objectivity, systematicity, and scientific rigor, this study identifies 5 dimensions: data quality, platform quality, platform service quality, user-perceived usefulness, and ease of use. The data quality dimension is subdivided into the quality of intrinsic data attributes and extrinsic attributes, while platform quality is subdivided into platform performance and functional attributes. Combined with the literature research, specific assessment indicators were identified under each dimension.

#### Screening of Assessment Indicators

The Delphi method was used to construct the medical data assessment system. Thirteen cross-disciplinary experts (5 data assessment experts, 4 medical informaticians, and 4 clinicians) were invited to conduct 3 rounds of iterative review to screen the indicators based on a 5-level Likert scale (retention thresholds: mean ≥3.0 and SD <1.0). The data accuracy indicator was removed due to overlap with data integrity, and new indicators related to service confidentiality, assurance, and other medical-specific characteristics were added, resulting in a 5D, 35-indicator system, with complete definitions provided in Table 2 and Multimedia Appendix 1.

**Table 2.** Indicator framework for assessing the value of medical science data.

Dimension and source	Subdimension	Indicators
Data quality		
Saxena [8]	Quality of intrinsic data attributes	Number of datasets Data integrity Data comprehensiveness Data timeliness Data authenticity
Zhang and Trace [24]	Quality of data external attributes	Data consistency Machine readability Format openness Data understandability
Platform quality		
Wu et al [26]	Quality of platform performance attributes	System stability System security System responsiveness System compatibility Interface friendliness Linguistic diversity
Martin et al [27]	Platform functional attributes	Platform infrastructure Platform overview function Platform guidance function Data access function Results display function Comprehensiveness of functions
Platform service quality		
Azeroual et al [28]	Quality of platform services	Service interactivity Service personalization Service accessibility Service confidentiality Service assurance Search comprehensiveness
User-perceived usefulness		
Imran and Ahmad [29]	User-perceived usefulness	Relevance Usefulness Uniqueness Novelty
User perceived ease of use		
Imran and Ahmad [29]	User perceived ease of use	Findable Accessible Interoperable Reusable

### Empirical Analysis

#### Sample Platform Selection

China’s open platforms for medical and scientific data boast several advantages, including “large data volume, diverse data sources, and high data quality.” However, they also face challenges such as “an overemphasis on data security, limited data sharing, and reliance on a single data-providing organization.” The sample platforms were screened from authoritative websites such as Re3data.org and FAIRsharing. The selection criteria required that the platforms be open and stable, provide basic data, regularly update content, offer comprehensive functionality, and demonstrate mature construction. The selection process for the sample platform is shown in the Multimedia Appendix 2. Table 3 lists the selected platforms, and data collection was conducted using Octopus (Shenzhen Digital Broad Information Technology Co).

**Table 3.** Overview of the 10 sample platforms selected for this study.

Nation	Name of platform	Developer	Data resource
China	National Population Health Sciences Data Center (NCMI)	Institute of Medical Information, Chinese Academy of Medical Sciences	Population health science data
China	GigaDB Database	Beijing Institute of Genomics, Chinese Academy of Sciences	Biological and biomedical field data
China	GSA^a^ for Human	National Genomics Data Center (NGDC)	Raw data on human genetic resources genomics
United States	National Addiction and HIV Data Archive Program (NAHDAP)	Inter-University Consortium for Political and Social Research (ICPSR)	Data from research related to drug addiction and HIV
United States	PhysioNet	Massachusetts Institute of Technology (MIT)	Physiological and clinical data
Multinational	MGnify	European Bioinformatics Institute (EMBL-EBI)	Microbiome data and macrogenomic data
United States	OpenNeuro	Stanford University	Brain imaging datasets
United States	Immunology Database and Analysis Portal (ImmPort)	National Institute of Allergy and Infectious Diseases (NIAID)	Clinical and basic research data
Multinational	BioImage Archive	European Bioinformatics Institute (EMBL-EBI)	Bioimaging data
Multinational	EBRAINS	Human Brain Project (HBP)	Brain research data

a GSA: Genome Sequence Archive.

#### Calculation of Indicator Scores

The indicators can be divided into three categories: (1) simple indicators, such as the number of datasets and data completeness, can be determined by simple calculations and are suitable for manual checking, 0‐1 assignment, and mathematical statistics; (2) text-based indicators, such as data comprehensiveness and comprehensibility, necessitate the processing of textual information, such as data categories and data summaries, and are suited for data crawling and text analysis; (3) subjective indicators, such as interface friendliness and relevance, are based on users’ subjective feelings and can be calculated using the illumination interview approach. The formulas and results of each indicator are shown in the Multimedia Appendix 3.

#### Indicator Reliability Tests

The ICC can be used to determine consistency. In this paper, both the differences between different assessment indicators and the differences between different platforms are considered, while systematic errors are not considered. The output results from the Statistical Product and Service Solutions Automatically Using Software are shown in Table 4.

Because the evaluation index scores are processed in advance, the “average measure ICC(C,K)” is used, with an ICC value ranging from 0 to 1. ICC values of more than 0.40 typically indicate good consistency. Table 4 reveals that the final ICC value is 0.489, which is greater than 0.4, indicating that the evaluation’s consistency is good and the indicator is reasonable.

**Table 4.** Results of correlation coefficients within intraclass correlation coefficient groups.

Bidirectional mixing and randomization consistency	ICC^a^ (95% CI)
Single metric ICC(C^b^,1^c^)	0.027 (−0.003 to 0.137)
Average metric ICC(C,K)^d^	0.489 (−0.101 to 0.848)

a ICC: intraclass correlation coefficient.

b C denotes consistency.

c 1 denotes a single measure.

d K denotes an average measure.

#### Calculation of Indicator Weights

The entropy weight approach is appropriate for dealing with the problem of multi-indicator empowerment because it can handle the interplay between various elements while also considering the correlation between different indicators. Its specific steps include (1) data standardization, (2) calculation of the ratio of each indicator in each scheme, (3) calculation of the information entropy of each indicator, (4) calculation of the weight of each indicator through the information entropy value, and (5) calculation of the comprehensive score of each scheme. Multimedia Appendix 4 shows the weights of the complete indicators derived from the entropy weight method.

### Ethical Considerations

This study did not require formal ethics committee review, as it exclusively analyzed publicly accessible platform interfaces, technical documentation, and aggregated system-level characteristics, without the collection of identifiable human data. This exemption aligns with the National Health Commission of China Regulations, Article 24, which states: “Ethics review may be waived for studies using publicly available data or records where individuals cannot be identified, and the research does not interact with human participants.” It also aligns with the JMIR policy on nonhuman participants research, which states: “Ethics approval is not required for studies evaluating technology systems, public documentation, or organizational practices where human participants are neither involved nor identifiable.”

## Results

### Comprehensive Analysis

To determine the optimal and worst solutions, the positive and negative ideal solution distances and relative proximity were calculated, as shown in Table 5. From Table 5, it can be seen that the National Population Health Sciences Data Center (NCMI; Institute of Medical Information, Chinese Academy of Medical Sciences) has the highest data value, which is close to the ideal level and serves as a valuable benchmark for other platforms.

**Table 5.** Technique for Order Preference by Similarity to Ideal Solution (TOPSIS) evaluation calculations.

Platform	Distance to positive ideal solution, D^+^ (unit:fraction)	Distance to negative ideal solution, D^-^ (unit:fraction)	Relative proximity, C (dimensionless)	Sort result
NCMI^a^	17.384	22.253	0.561	1
GigaDB	25.848	5.021	0.163	8
GSA^b^ for Human	22.135	14.685	0.399	3
NAHDAP^c^	27.571	3.289	0.107	10
PhysioNet	25.939	6.400	0.198	7
MGnify	18.945	15.405	0.448	2
OpenNeuro	24.785	12.671	0.338	5
ImmPort	24.143	9.990	0.293	6
BioImage Archive	26.479	4.795	0.153	9
EBRAINS	23.127	14.120	0.379	4

a NCMI: National Population Health Sciences Data Center.

b GSA: Genome Sequence Archive.

c NAHDAP: National Addiction and HIV Data Archive Program.

The weighted formula was used to calculate the scores and composite scores for the different dimensions of data value for each platform:


(1) $$Score\left(P_{ij}\right)=\sum w_{k}X_{k}$$

(2) $$Score\left(P_{i}\right)=\sum_{j=1}^{7}w_{j}Score\left(P_{ij}\right)$$

**Figure 5.**
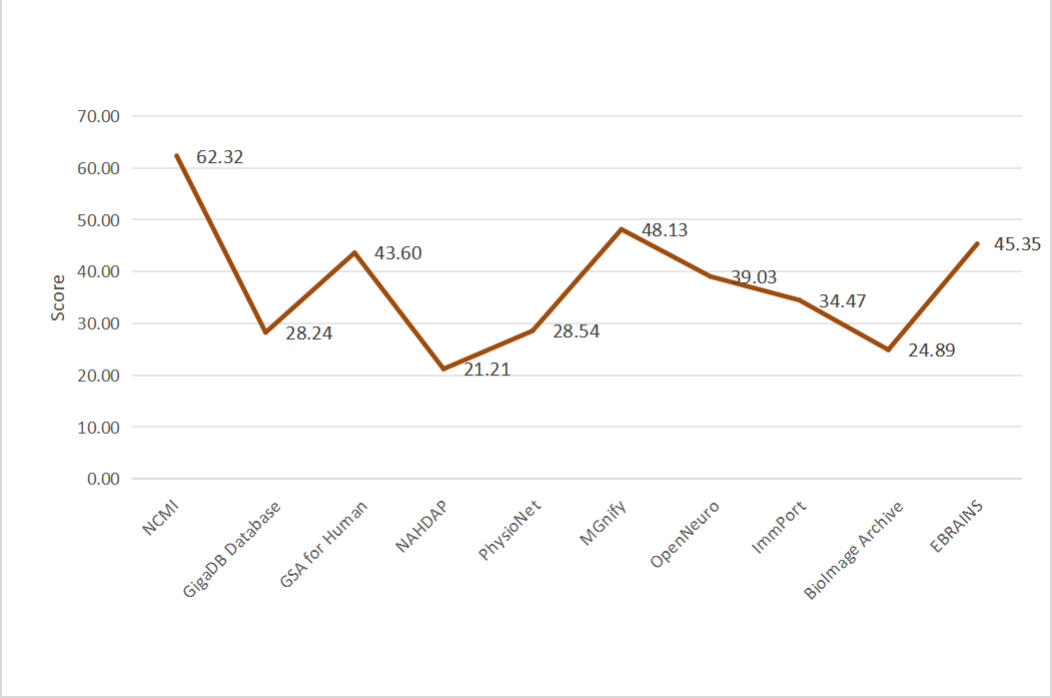
Line graph of data value dimension scores for each platform. GSA: Genome Sequence Archive; NAHDAP: National Addiction and HIV Data Archive Program; NCMI: National Population Health Sciences Data Center.

Where i denotes the ith platform, j the jth dimension, and k the kth indication. Score (Pᵢⱼ) reflects the data value score of the jth dimension of the ith platform, as well as the platform’s overall data value score. Figures5  6 show the differences between each platform’s data value by dimension and their comprehensive scores.

As shown in Figures5  6, there are large differences in data value across the dimensions of intrinsic attribute quality, extrinsic attribute quality, platform service quality, and perceived usefulness. The average score of the platforms is 37.579, with NCMI, MGnify (European Bioinformatics Institute), EBRAINS (Human Brain Project), GSA for Human (National Genomics Data Center), and OpenNeuro (Stanford University) scoring higher than the average, reflecting good overall performance.

**Figure 6.**
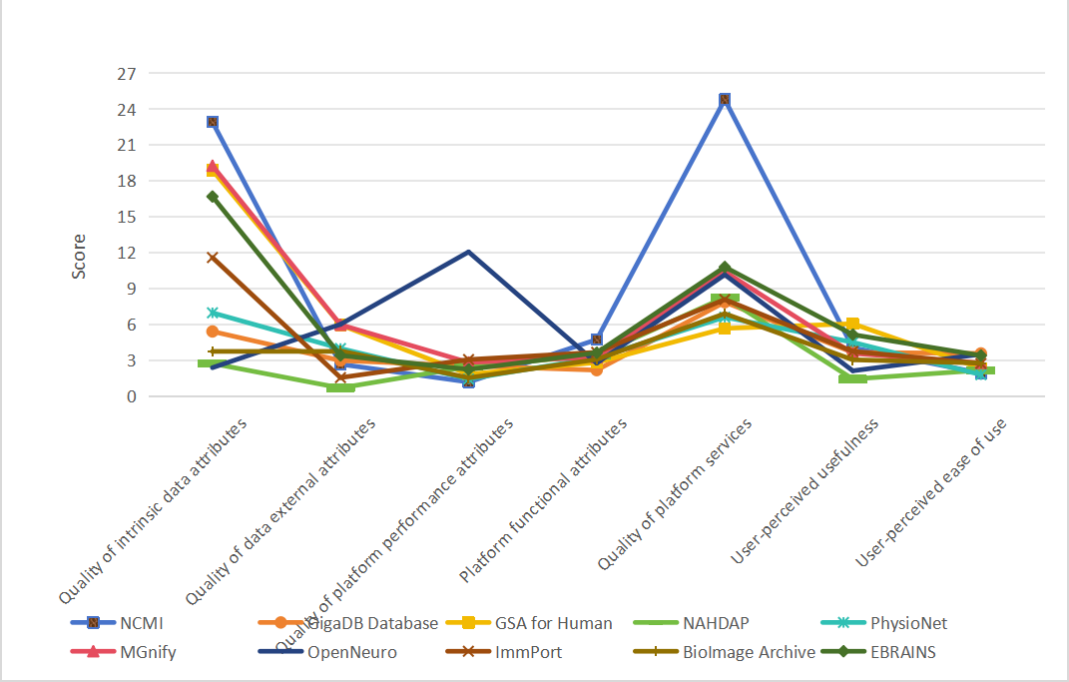
Line graph of the composite data value score of each platform. GSA: Genome Sequence Archive; NAHDAP: National Addiction and HIV Data Archive Program; NCMI: National Population Health Sciences Data Center.

### Model Validation

The evaluation reveals that (1) Chinese platforms demonstrate data quality strengths but require improvements (eg, NCMI shows slow updates and low availability of machine-readable format; GSA lacks completeness in data attributes/types), (2) platform functionality favors NCMI for comprehensiveness, while OpenNeuro leads in responsiveness, (3) service quality analysis shows NCMI’s advantages in personalization and search capability; however, all platforms need enhancements in service assurance and confidentiality, (4) GSA excels in content novelty/relevance despite overall low data utility across platforms, and (5) GiagDB demonstrates strong usability despite partial FAIR compliance. The model successfully identifies critical gaps in China’s platform data management and proposes targeted optimization pathways, thereby validating its scientific rigor.

## Discussion

### Principal Findings

This study establishes a novel medical data valuation framework through a 3D (data-platform-user) analysis, integrating service privacy metrics. By combining qualitative and quantitative methods, the hybrid approach reduces subjectivity bias, generating an evaluation model that enables data value quantification and personalized management strategies.

Comparing the results of government data value assessment, the following conclusions can be drawn: (1) indicators related to the data itself, such as the number of datasets, data timeliness, and machine readability, as well as indicators such as system responsiveness and service interactivity, are considered very important, (2) compared with government data, medical science data is private and scientific, so the weight of indicators such as service security, service confidentiality, accessibility, and reusability are more prominent, and (3) in the process of data value realization, the data itself have potential value, but their actual realization requires further development, so indicators related to platform quality and platform service quality, such as search comprehensiveness and service personalization, are also crucial.

### Model Application

#### Optimize the Process of Assessing the Value of Platform Data

**Figure 7.**
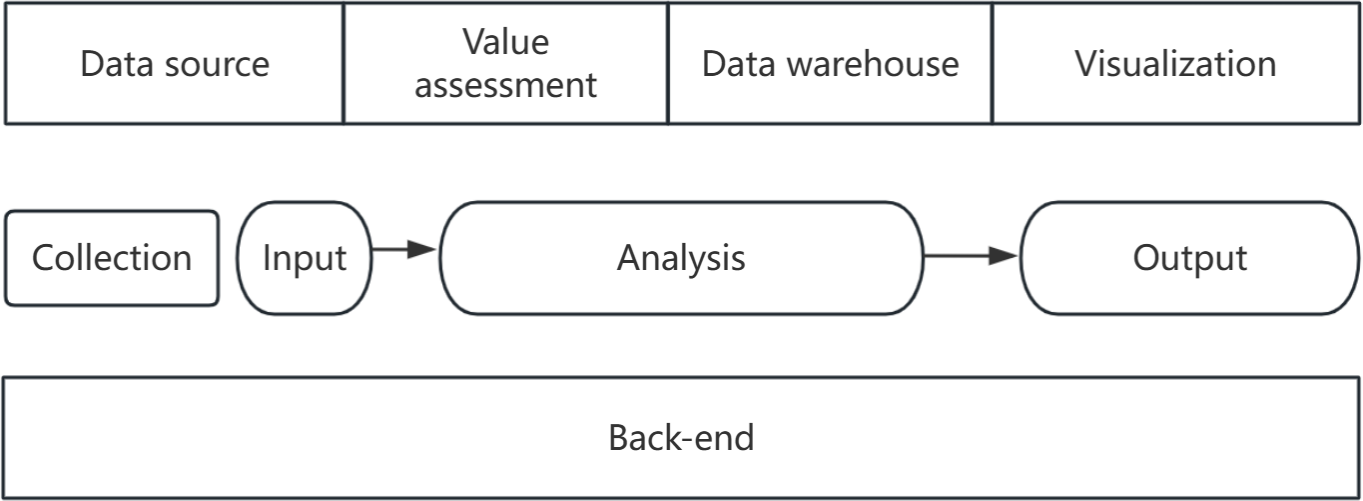
Architecture of the data value assessment system.

1. To assist platforms in efficiently assessing data value and formulating management strategies, this study proposes two practical solutions based on the established evaluation index system: (1) designing a universal self-assessment tool, leveraging the international Confederation of Open Access Repositories (COAR) community framework to enable convenient self-evaluation; and (2) developing an intelligent assessment system that enhances evaluation efficiency by integrating automation technologies with human intervention. These solutions synergistically advance data value assessment from theoretical frameworks to practical implementation.
2. Design of a generic self-assessment tool: the COAR, an international association that brings together repositories from around the world to build a global network of knowledge repositories, has developed a global COAR Community of Good Practice for Repositories framework designed to help repositories in different regions and of different types evaluate and improve their practices [30]. In November 2023, the COAR Asia OA Webinar, based on the COAR community framework, proposed the survey toolkit—a self-assessment tool for repository development that asks repositories to answer 60 questions to assess their practices and identify areas for improvement. Repositories that provide affirmative responses to all “Essential” questions are awarded a digital badge, which can be displayed on their websites [31]. Drawing inspiration from these examples, this study can also propose a data value self-assessment tool, which will undergo iterative updates and refinements based on feedback and usage.
3. Development of an automated assessment system: creating automated and semiautomated assessment tools can improve the accuracy and efficiency of assessments. Blacketer et al [32] created the data quality dashboard, an open-source R package to evaluate the quality of observational health care data and indicate potential data quality issues by merging the Data General Model with established data quality assessment methodologies. The architecture of the automated data value assessment system (Figure 7) should cover the modules of input, analysis, output, and back-end, and the assessment of the implementation process should include several aspects: the data source, data quality assessment model, and data warehouse. Therefore, analyzing the data value of the platform should be done systematically and configured with automated assessment procedures and methods, tools, and software.

The automated assessment of data value faces a double challenge: at the technical level, it needs to break through data dependency, cross-domain generalization bottlenecks, and algorithmic interpretability defects; at the ethical level, it must construct a privacy-informed dynamic balancing mechanism that controls leakage risk through differential privacy and federated learning, and embeds fairness checking modules to eliminate group bias.

#### Multidimensional Improvement of the Data Value of the Platform

To advance medical data platforms holistically, this study proposes a multifaceted enhancement strategy spanning four critical dimensions: implementing dual-track data quality control (source integration and FAIR-aligned standardization), upgrading platform performance functionality through technical optimization and value-visualization tools, establishing a tripartite privacy governance framework (organizational-management-technical), and constructing user-centric service ecosystems with demand-driven customization and collaborative training.

A dual strategy is needed to improve data quality. First, a mechanism should be established for data aggregation from multiple sources, including integration of data resources from research institutions, development of unified classification standards, and implementation of update cycles to maintain data currency. Second, data presentation format should be standardized by mandating the use of machine-readable formats, ensuring metadata completeness, and aligning attribute descriptions with the FAIR principles. Technically, intelligent quality control tools can be integrated, such as an RNA-Seq data cleaning algorithm [33], to automatically filter contaminated data.

The quality of the platform must be improved in both directions through performance and functionality: the technical side adopts resource compression algorithms and multilevel content delivery network caching to ensure that page loading time is reasonable; the functional side builds a data value visualization system (dynamic leaderboards/timing trend charts) and integrates data challenges and health apps to improve the usage rate. The platform needs to expand higher-order functional modules (eg, thematic data aggregation, blockchain traceability verification, and knowledge graph mining), build a full-cycle service system (training-consultation-feedback closed-loop), and improve the effectiveness of data usage through intelligent operation and maintenance optimization.

In terms of data services, the platform can draw on the Tencent Foraging Open Experimental Platform (jointly developed by Tencent Cloud and a team from Tencent's Medical AI Lab) to build a comprehensive intelligent service system for medical imaging research application scenarios. This includes data management and labeling, algorithm training, testing, and application. In addition, medical data privacy protection needs to build an organization-management-technology synergistic system: set up an ethical review committee at the organizational level and formulate a GDPR (General Data Protection Regulation)-compatible privacy agreement, implement k-anonymization and homomorphic encryption technology at the management level, and deploy a federal learning framework at the technical level to achieve data availability and invisibility. Given the existing problems, such as the disconnection between privacy algorithms and scenarios, and the shortcomings of a single level of protection, it is recommended that we borrow the concept of the United Kingdom’s “five safe” to build a trinity of protection systems for “data-people-environment,” and synchronize the development of a multilayered architectural platform similar to the MNSSp3, so that we can balance the value of medical research with individual privacy rights and interests [34]. The platform needs to build a user-centered service system. First, it should build a demand portrait based on collaborative filtering algorithms, collect user feedback on time, dynamically adjust the data opening strategy, realize the accurate push of high-value data, establish a grading model of user capabilities, and provide differentiated data service interfaces. Second, the platform should unite with academic institutions to create a modular training system, integrate multidimensional education scenarios such as workshops, internet-based communities, and data competitions, break through the traditional one-way training model, and improve users’ data reuse capabilities. It can draw on the World Health Organization (WHO) to create automated tools and regional customized training experiences [35].

### Limitations

This study acknowledges three principal methodological constraints that warrant consideration when interpreting the findings:

1. Indicator subjectivity: the construction of the indicator system and the scoring of certain indicators are more subjective, which may introduce assessment bias. The expert survey method was used to discuss and screen the indicators, and the ICC method was used to test the consistency of the assessment; the ICC value is 0.489, indicating that the consistency is at a medium level. This may be due to the differences among evaluators, overly complex measurement dimensions, or a small sample size. This issue can be improved by clarifying the scoring criteria, revising the scale, and increasing the sample size.
2. Sample coverage: the current empirical evidence covers 10 platforms. Although the number of sample platforms selected is relatively limited, it covers mainstream medical data types and has a certain selection basis (refer to Multimedia Appendix 2 for details). A follow-up study is planned to expand the number of sample platforms and include European medical science data platforms.
3. Limitations of value dimension: data value assessment is carried out along the mechanism of “value perception and identification→value realization→value assessment→data governance.” However, this paper focuses on the assessment of perceived value and does not involve the mechanism of value realization. The next step is to construct a data trust assessment framework, develop value stream analysis tools, and explore collaborative governance models.

### Comparison With Prior Work

At the theoretical level, this study broadens the perspectives, objects, and methods of data value assessment research and refines the strategies for value enhancement of medical science data on open platforms. At the practical level, the assessment model developed in this study can not only help platforms reasonably quantify the potential value of data, identify key factors in the formation of data value, discover deficiencies in data collection and organization, formulate data investment decisions and openness plans, and promote the effective allocation of data resources; it can also help users recognize the potential benefits of the data on the platforms and better use the data resources in their data activities, and help countries to understand the benefits of open medical science data to optimize data regulations and systems.

### Conclusions

This study focuses on assessing the value of medical science data from the perspective of platforms, and the results of the study include (1) a data value assessment index system with 5 dimensions and 35 indicators was created by using ISSM, TAM, and the theory of perceived value of consumers, which is scientific, (2) 10 open platforms were selected, data were collected through Octopus and empirically examined using the entropy weight Technique for Order Preference by Similarity to Ideal Solution method, and the assessment model was established and scientifically verified, and (3) the practical application of the proposed model can not only optimize the data value assessment process, but also enhance the data value of the platform from multiple dimensions.

## Supplementary material

10.2196/63544 Multimedia Appendix 1 Indicator framework for assessing the value of medical science data.

10.2196/63544 Multimedia Appendix 2 The selection process for the sample platform.

10.2196/63544 Multimedia Appendix 3 The calculation methods and results of the 35 evaluation indicators.

10.2196/63544 Multimedia Appendix 4 Summary of the results of the entropy method of calculating weights.

## Abbreviations

COAR: Confederation of Open Access Repositories
FAIR: Findable, Accessible, Interoperable, and Reusable
GDPR: General Data Protection Regulation
ICC: intraclass correlation coefficient
ISSM: information systems success model
NCMI: National Population Health Sciences Data Center
TAM: technology acceptance model
WHO: World Health Organization
