# Supplementary material for: Medical Science Data Value Evaluation Model: Mixed Methods Study
Source: JMIR Med Inform. 2025 Aug 21;13:e63544. doi: 10.2196/63544 (PMC12369987; doi:10.2196/63544)
Supplement: Multimedia Appendix 1 [file medinform-v13-e63544-s001.docx]

# Multimedia Appendix 1

Indicator framework for assessing the value of medical science data

| Dimension | Subdimension | Indicator | Interpretation of indicators |
| --- | --- | --- | --- |
| Data  Quality | Quality of intrinsic data attributes | Number of data sets | Number of datasets on the platform |
|  |  | Data integrity | Breadth, depth, and structural integrity of data |
|  |  | Data comprehensiveness | Comprehensive coverage of data topics |
|  |  | Data timeliness | Data can be updated in a timely manner |
|  |  | Data authenticity | Data is authentic and presented in its original form |
|  | Quality of data external attributes | Data consistency | Data is presented in a consistent format |
|  |  | Machine Readability | Data format is machine-recognizable |
|  |  | Format openness | Data are presented in an open format |
|  |  | Data understandability | Data descriptions are concise and easy to understand |
| Platform quality | Quality of platform performance attributes | System Stability | Platform links are valid and data are accessible |
|  |  | System security | Secure platform and no non-relevant pop-ups |
|  |  | System responsiveness | The system can respond quickly to user requests |
|  |  | System Compatibility | The platform supports for different operating systems and browsers |
|  |  | Interface friendliness | The interface is well laid out and visually appealing |
|  |  | Linguistic diversity | The platform is available in multiple languages |
|  | Platform Functional Attributes | Platform infrastructure | Policy development and organizational foundations |
|  |  | Platform overview function | Overview of data presented by the Platform to users |
|  |  | Platform guidance function | The platform provides guidance functions for users |
|  |  | Data access function | Data browsing and access functions |
|  |  | Results display function | Various data results shown by the Platform |
|  |  | Comprehensivenesso f functions | Diverse data functions provided by the platform interface |

| Dimension | Subdimension | Indicator | Interpretation of indicators |
| --- | --- | --- | --- |
| Quality of platform services | / | Service interactivity | Interactive communication services provided by the Platform |
|  |  | Service personalization | Personalized services provided by the Platform to users |
|  |  | Service accessibility | Ancillary services provided by the Platform to users |
|  |  | Service confidentiality | Services provided by platforms to protect user privacy |
|  |  | Service assurance | Authority of the platform (service) |
|  |  | Search comprehensiveness | Total number of advanced search term functions by platform |
| User-perceived usefulness | / | Relevance | Retrieve data relevant to the user's research topic |
|  |  | Usefulness | Benefits of user access to and use of data |
|  |  | Uniqueness | Users perceive data as unique and unduplicated |
|  |  | Novelty | Users perceive data sources or processes as innovative |
| User Perceived Ease of Use | / | Findable | Users can discover data with identifiers and metadata |
|  |  | Accessible | Users can access data through registration and agreements |
|  |  | Interoperable | Users can manipulate data with FAIR word lists, etc. |
|  |  | Reusable | Users can reuse data based on usage agreements |
