# Supplementary figures and images for: Medical Science Data Value Evaluation Model: Mixed Methods Study
Source: JMIR Med Inform. 2025 Aug 21;13:e63544. doi: 10.2196/63544 (PMC12369987; doi:10.2196/63544)

#
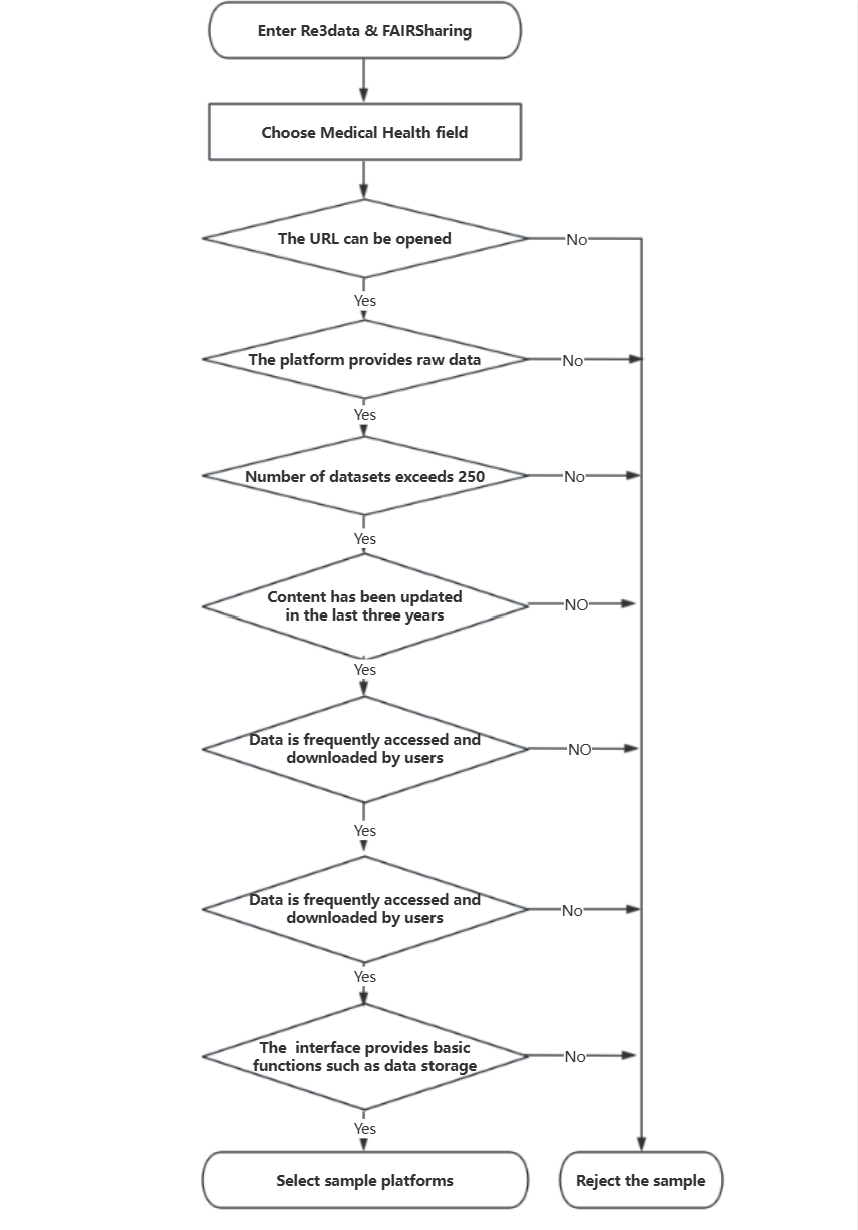
Multimedia Appendix 2

The selection process for the sample platform

Supplement: Multimedia Appendix 2 [file medinform-v13-e63544-s002.docx]
