# Supplementary material for: Medical Science Data Value Evaluation Model: Mixed Methods Study
Source: JMIR Med Inform. 2025 Aug 21;13:e63544. doi: 10.2196/63544 (PMC12369987; doi:10.2196/63544)
Supplement: Multimedia Appendix 3 [file medinform-v13-e63544-s003.docx]

# Multimedia Appendix 3

The calculation methods and results of the 35 evaluation indicators are as follows:

(1) Number of data sets

The number of platform datasets can reflect the platform data load; the larger the data volume, which contains more useful information, the greater the value of the data. As of August 24, 2023, the data volume of each platform for manual checking was 17,534, 2,387, 3,594, 586, 275, 4,697, 896, 649, 2,275, and 978, and the score of this indicator calculated by the extreme value method was 100, 13.61, 20.50, 3.34, 1.57, 26.79, 5.11, 3.70, 12.98, and 5.58.

(2) Data integrity

Data integrity includes the breadth, depth, and structural integrity of data, as well as metadata and data file integrity. Drawing on the data integrity measure proposed by Azeroual et al. [6] , the metric is defined as:

$V_{Data Quality}P_{Completeness}=1-\frac{NuLL}{Number of checked units}$ (1)

The denominator represents the number of all attributes of the sampled data and the number of NULL missing attributes. The common integrity attributes are DOI, title, creator, release time, data size, data format, data version, data description, keywords, and data type. 100 pieces of data from each platform were randomly selected and manually checked, and the scores for this indicator were calculated using the formula: 91.6, 96.3, 69.8 ,100, 84.2, 55.2, 83.5, 88.7, 67.1, and 86.2.

(3) Data comprehensiveness

Data comprehensiveness refers to the richness of data content and themes, and the coverage of data themes directly determines the breadth and depth of data openness, thus affecting its value. With reference to the original data classification of the platform, the index score is determined by combining the micro-word cloud as follows: 100, 45.46, 40.91, 27.27, 40.91, 25, 11.36, 29.55, 13.6, 45.46.

(4) Data timeliness

Data timeliness, i.e., the age of the data, refers to the ability of the data to be updated in a timely manner, indirectly reflecting the truthfulness and reliability of the data, which can be defined:

$V_{data quality}(P_{Ti\mathrm{meliness}})=\frac{day(D_{e}-D_{u})}{day(D_{u}-D_{s})}\times100\%$ (2)

De represents the current survey date, Du represents the date of the most recent update, Ds represents the date of the first release, PTimeliness represents the timeliness of the platform data, the smaller the value the better the data timeliness, and has the following calculation rules:

$V_{\mathrm{data}\mathrm{quality}}(P_{\mathrm{Timeliness}})=\left\{ \begin{aligned} 0，D_{s}<D_{u}=D_{e}，\mathrm{best} \\ （0，1），D_{s}{<D}_{u}<D_{e}，eligible \\ 1，D_{s}=D_{u}\leq D_{e}，pointless \\ （1，+\infty），D_{s}<D_{u}<D_{e}，unqualified \end{aligned} \right.$ (3)

By sampling and checking the date of publication and date of modification of each platform's dataset, the following scores were calculated for this indicator: 4.43, 5.25, 98.65, 4.58, 34.47, 98.65, 5.21, 69.59, 4.70, 100.

(5) Data authenticity

Authenticity means that the data are real and reliable, and the data are kept in their original form. By means of the survey platform data production method, the score of this indicator is calculated as 95.80, 94.85, 100, 100, 100, 100, 100, 100, 100, 100.

$V_{data quality}(P_{Data authenticity})=\frac{K_{i}}{\mathrm{Max}{(K}_{i})}\times1$00% (4)

(6) Data consistency

Data consistency means that data are logically consistent, that common concepts, classifications, and representations are used throughout the system, and that any differences are within the bounds of interpretability. By checking the maximum number of datasets presented in a consistent format across platforms, the indicatorwas assigned a value of 100, 100, 100, 100, 100, 100, 100, 100, 62.20, 100.

(7) Machine Readability

Machine readability refers to whether the platform data is presented in a machine-readable format; the higher the machine readability, the more convenient it is for users to access the data. The machine readability calculation requires first defining the Boolean function:

$\mathrm{is}MachineReadable(k_{i})=\left\{ \begin{aligned} {0， k}_{i}is not in the list of machine-readable formats \\ 1，K is in the list of machine-readable formats \end{aligned} \right.$ (5)

ki stands for the data format of the ith data; the list of machine-readable formats can be borrowed from the OpenDataMonitor's machine-readable formats [30].

Also defined:

$V_{Data Quality}(P_{\mathrm{MachineReadable}})=\frac{\sum_{i=1}^{N} \mathrm{Metric}（k_{i},isMachineReadable）}{N}\times100\%$ (6)

PMachineReadable stands for platform data machine-readable; N stands for the number of platform data. If the data format represented by Ki can be found in the list of machine-readable formats, equation (4) returns 1. Through sampling and manual checking, the scores of this indicator are: 41, 44, 100, 0, 62, 100, 100, 17, 61, and 53.

(8) Format openness

Openness of format refers to data being presented in an open format that is easily and freely accessible to users. A list of descriptions of open formats of data from sampled data, collected data file formats, and comparisons of open data monitors yielded the following scores for this indicator for each platform: 50, 94, 100, 100, 98, 100, 100, 93, 94, and 80.

(9) Data understandability

Data understandability refers to whether the data is easy to understand and interpret and can be defined as:

$P_{U\mathrm{nder}\mathrm{standability}}=\frac{\sum_{i=1}^{N} \mathrm{Metric}（k_{\left\{ \left. \mathrm{abstract} \right\} \right.i},Flesch）}{N}\times100\%$ (7)

$k_{\left\{ \left. \mathrm{abstract} \right\} \right.i}$ represents the data description of each platform, and N represents the amount of platform data. Except for the dataset description of NCMI, which involves both Chinese and English, the dataset descriptions of the other platforms are in English and are calculated using textstat provided by Python and the built-in formula textstat.flesch_reading_ease(abstract), which results in the following scores for this indicator: 33.59, 27.47, 26.42, 29.66, 38.49, 30.61, 41.20, 27.83, 23.41, and 28.90.

(10) System Stability

System stability is also referred to as link validity or accessibility. It determines whether users can successfully obtain and use data, and can be defined as the ratio of the number of links that users can effectively access to the total number of queries:

$P_{S\mathrm{ta}\mathrm{bility}}=\frac{\sum_{i=1}^{N} \mathrm{Metric}（k_{i},isE\mathrm{rr}\mathrm{orCode}）}{N}\times100\%$ (8)

The numerator represents the number of valid links on the platform, and N represents the total number of queries. Randomly clicking on 100 dataset links for each platform, it is found that all can be opened normally, so the system stability index of each platform is assigned a value of 100.

(11) System security

Randomly clicking on 100 data sets for each platform, it was found that no non-relevant pop-up window appeared in the process of opening, so the system security index of each platform was assigned a value of 100, respectively.

(12) System responsiveness

System responsiveness refers to the time it takes for the system to return the appropriate data to the user request from the time it is accepted to the time the processing is completed. Sucuri Load Time Tester was utilized to test the average loading time of each web page, and the index scores were calculated as 5.61, 17.57, 8.18, 16.87, 8.13, 19.9, 100, 21.99, 8.82, and 14.98 by using extreme value method.

(13) System Compatibility

System compatibility means that the platform supports various operating systems and browsers. By checking the operation of the platform on Firefox, Chrome, Edge, Safari, and IE browsers on Windows, MacOS, and Linux systems, the index score is calculated as 100, 100, 90.91, 100, 90.91, 90.91, 90.91, 100, 100, 90.91.

(14) Interface friendliness

Interface friendliness means that data can be presented in an appropriate form, the interface is clear and simple, the data are well organized, and it has good visual effects and satisfies user aesthetics. Invite a number of master's degree students and doctoral students to score the interface friendliness of each platform according to the prompts, take the average score, and use the extreme value method to calculate the score of this index: 100, 79.73, 77.03, 89.19, 70.27, 91.89, 82.43, 90.54, 86.49, 78.38.

(15) Linguistic diversity

Suitable interface languages help assist users in navigating and understanding the data, thus making it easier to access and use the data. Through manual checking, it was found that only GSA for Human provides both Chinese and English, while the other platforms only provide English, so the value of this indicator was assigned as: 50, 50, 100, 50, 50, 50, 50, 50, 50, 50.

(16) Platform infrastructure

Platform infrastructure refers to data-related regulations, policies, and organizational leadership. Regulations and policies refer to whether the platform publishes data-related policies (at the national and institutional levels), and organizational leadership refers to whether the platform makes public the contact information of the leading group and relevant departments, with the scores for this indicator being: 100, 75, 100, 100, 75, 75, 75, 100, 75, 75.

(17) Platform overview function

Platform overview function refers to the data overview presented by the open data platform, including data update status, data download status, the latest relevant information, and visualization display. By manually checking the types of platform overview functions of each platform, it is determined that the score of this indicator for each platform is 100, 25, 75, 75, 50, 75, 50, 75, 25, 50.

(18) Platform guidance function

Platform guidance function refers to the open data platform providing users with guidance functions to facilitate users' data search, including categorized navigation function, search function, sorting function, and inter-platform links. By checking the types of guidance functions of each platform, it is determined that the score of this indicator is 100, 75, 75, 100, 100, 100, 100, 100, 100, 75.

(19) Data access function

Data access function means that the open data platform provides relevant functions to facilitate users' browsing and access to target data, including data preview, open data catalog, local access, and hierarchical classification access. By checking the types of data access functions of each platform, the score of this indicator was determined to be 75, 75, 75, 100, 75, 75, 75, 50, 50, 75.

(20) Results display function

The results display function refers to the open data platform showing the public the various types of results produced by users after utilizing data for research and development, including featured databases, data projects, data tools, and data competitions. By checking the types of results on each platform, the score of this indicator is determined as 100, 50, 25, 50, 75, 50, 50, 75, 100, 100.

(21) Comprehensiveness of functions

Comprehensiveness of functions means that the platform has comprehensive functions that can meet the different needs of users, including data remittance, storage, release, management, browsing, retrieval, download, tool provision, analysis and visualization, and user training. The indicator scores of 100, 70, 100, 90, 90, 100, 100, 100, 100, 100 were determined by manual checking.

(22) Service interactivity

Service interactivity means that the platform helps users to collaborate, communicate, and share, and platform interactive functions such as dataset evaluation, data request, suggestion feedback, data error correction, and sharing and dissemination. By looking at the types of communication and feedback functions of each platform, it was determined that the scores for this indicator for each platform were: 80, 80, 60, 40, 60, 40, 60, 20, 60, 100.

(23) Service personalization

Service personalization refers to the platform's personalized services to attract and retain users, including personalized accounts, personalized integration, dataset subscription or collection, intelligent services, and dataset recommendation. By checking the types of personalized services on each platform, the score of this indicator is determined as: 100, 20, 20, 20, 20, 20, 60, 20, 20, 60.

(24) Service accessibility

Service accessibility refers to the platform's provision of auxiliary services to help users solve problems in the use of the system, and such services include online consultation, help documentation, and so on. By checking the types of auxiliary services provided by each platform, the scores for this indicator were determined as follows: 71.43, 71.43, 28.57, 71.43, 57.14, 85.71, 71.43, 100, 85.71, 100.

(25) Service confidentiality

Service confidentiality refers to the platform's safeguards for data security and user privacy, which are crucial to the value of medical data. In this paper, we refer to the steps taken by ICPSR, an international authoritative organization, to maintain data confidentiality [31]. The platform's management, technical, and policy means adopted to determine the content of this indicator include: reviewing all datasets to assess disclosure risk; making necessary changes to data to reduce disclosure risk; restricting access to datasets; training staff to ensure data security through organizational measures; sharing risk assessment and mitigation strategies with data producers; implementing managerial and technical measures to ensure data privacy; and providing data privacy policies and agreements. The scores for this indicator are: 85.71, 57.14, 71.43, 100, 28.57, 28.57, 71.43, 28.57, 28.57, 57.14.

(26) Service Assurance

Service assurance refers to the platform information service having a certain guarantee; whether the platform is trustworthy or not is the key index to measure the assurance. By checking the CoreTrustSeal certification of each platform and the endorsement policy on FAIRsharing, the scores of this indicator are determined as: 50, 50, 0, 50, 50, 50, 50, 100, 50, 50.

(27) Search comprehensiveness

Comprehensiveness of retrieval refers to the ability of users to conveniently retrieve the required data by utilizing the retrieval functions provided by the platforms. By checking the total number of advanced search term functions of each platform, it was found that, with the exception of NCMI and MGnify, all the other platforms provide only one-box searches, and the scores for this indicator were determined as follows: 100, 10, 10, 10, 10, 40, 10, 10, 10, 10.

(28) Relevance

Relevance means that the data content and subject matter are of interest and need to the inquirer, in line with his or her research, and able to address his or her needs. Three keywords are randomly selected on each platform, and the relevance of individual topics is calculated with the help of keyword search and the formula “topic relevance = topic-related tuple/total tuple” and averaged to obtain the relevance index score for each platform: 92.46, 76.34, 100, 19.35, 100, 87.09, 17.7, 53, 38.84, and 97.22.

(29) Usefulness

Usefulness refers to the ability of the data to improve users' understanding of specific issues, stimulate new insights and actions, guide users to achieve their personal goals, and improve their health literacy. To measure the usefulness of the data, the bounce rate displayed on the website “similarweb” was utilized, and the positive treatment of the bounce rate resulted in a score for this indicator: 57.80, 56.40, 48.32, 41.25, 39.93, 46.94, 55.27, 87.20, 46.94, 100.

(30) Uniqueness

Uniqueness refers to the degree of duplication between data and published data, meaning that a particular data field, data record, or data set is not duplicated in the system. EXCEL provides “data duplicates” to determine whether the sample dataset exists on other platforms to determine the uniqueness of the user's perception of the data. The value of this indicator is assigned to 100.

(31) Novelty

Novelty refers to the degree to which data sources, methods, and processes are new to users. Generally speaking, data released at a later date may not be accessible or familiar to users, and a novelty indicator can be defined as the follow:

$P_{N\mathrm{ove}\mathrm{lty}}=\frac{Y}{X+Y}\times100\%$ （9）

X represents the amount of stock data on each platform and Y represents the amount of incremental data on the platform within one year; the larger the indicator, the more incremental data on the platform and the more novel the data. By querying the data release of each platform in the past year, the index score is determined: 8.31, 11.4, 47.36, 6.66, 17.45, 4.98, 18.42, 20.65, 25.63, and 17.38.

1. Findable

Findable is a prerequisite for data to be shared and utilized and refers to the ability of users to search for specific data. By investigating the identifier type, metadata format, metadata richness, metadata resource identifiers, and search engine discoverability of each platform and assigning values to them according to their rank, the score of this indicator was calculated as: 100, 100, 66.67, 100, 83.33, 91.67, 100, 100, 100, 100.

1. Accessible

Accessible refers to the need for users to access data after they have discovered it, including how to obtain authentication and authorization. Investigate the access registration, access protocol, user audit mechanism, and (meta-)data lifetime commitment of each platform and assign values to them according to the level, respectively, and calculate the score of this indicator as: 55.56, 100, 55.56, 44.44, 22.22, 88.89, 100, 77.78, 77.78, 88.89.

(34) Interoperable

Interoperable means that users can integrate data with other data for analysis, storage, and processing. Investigate the data file format, (meta-)data element description, and (meta-)data association of each platform, and assign values to them according to the level, and calculate the score of this indicator as: 33.33, 100, 55.56, 77.78, 55.56, 77.78, 66.67, 77.78, 55.56, 88.89.

(35) Reusable

Reusable refers to the reproduction of results with user-available data, and the value of data increases with the number of times it is reused. Investigating the clarity of license statement, standardization of statement format, clarity of restriction statement, clarity of traceability information, and standardization of traceability format of each platform, assigning values to them according to the level, and calculating the score of this indicator as: 50, 75, 75, 50, 75, 37.5, 100, 50, 75, 87.5.
