# Supplementary material for: Medical Science Data Value Evaluation Model: Mixed Methods Study
Source: JMIR Med Inform. 2025 Aug 21;13:e63544. doi: 10.2196/63544 (PMC12369987; doi:10.2196/63544)
Supplement: Multimedia Appendix 4 [file medinform-v13-e63544-s004.docx]

# Multimedia Appendix 4

Summary of the results of the entropy method of calculating weights

| items | Information entropy value | Information utility value | Weighting factor |
| --- | --- | --- | --- |
| Number of data sets | 0.6969 | 0.3031 | 17.68% |
| Data integrity | 0.9940 | 0.0060 | 0.35% |
| Data comprehensiveness | 0.9257 | 0.0743 | 4.33% |
| Data timeliness | 0.7696 | 0.2304 | 13.44% |
| Data authenticity | 0.9999 | 0.0001 | 0.00% |
| Data consistency | 0.9966 | 0.0034 | 0.20% |
| Machine Readability | 0.9095 | 0.0905 | 5.28% |
| Format openness | 0.9934 | 0.0066 | 0.39% |
| Data understandability | 0.9939 | 0.0061 | 0.35% |
| System Stability | 1.0000 | 0.0000 | 0.00% |
| System security | 1.0000 | 0.0000 | 0.00% |
| System responsiveness | 0.8020 | 0.1980 | 11.55% |
| System Compatibility | 0.9995 | 0.0005 | 0.03% |
| Interface friendliness | 0.9979 | 0.0021 | 0.12% |
| Linguistic diversity | 0.9867 | 0.0133 | 0.78% |
| Platform infrastructure | 0.9956 | 0.0044 | 0.26% |
| Platform overview function | 0.9660 | 0.0340 | 1.98% |
| Platform guidance function | 0.9965 | 0.0035 | 0.20% |
| Data access function | 0.9923 | 0.0077 | 0.45% |
| Results display function | 0.9686 | 0.0314 | 1.83% |
| Comprehensivenesso f functions | 0.9978 | 0.0022 | 0.13% |
| Service interactivity | 0.9691 | 0.0309 | 1.80% |
| Service personalization | 0.9021 | 0.0979 | 5.71% |
| Service accessibility | 0.9822 | 0.0178 | 1.04% |
| Service confidentiality | 0.9552 | 0.0448 | 2.61% |
| Service Assurance | 0.9399 | 0.0601 | 3.51% |
| Search comprehensiveness | 0.7784 | 0.2216 | 12.92% |
| Relevance | 0.9455 | 0.0545 | 3.18% |
| Usefulness | 0.9791 | 0.0209 | 1.22% |
| Uniqueness | 1.0000 | 0.0000 | 0.00% |
| Novelty | 0.9169 | 0.0831 | 4.84% |
| Findable | 0.9970 | 0.0030 | 0.17% |
| Accessible | 0.9709 | 0.0291 | 1.70% |
| Interoperable | 0.9836 | 0.0164 | 0.96% |
| Reusable | 0.9829 | 0.0171 | 1.00% |
